# Mrs. Jennie E. Barker

**Published:** 1916-07

**Authors:** 


**Mrs. Jennie E. Barker** died in Buffalo, June 10. She was the widow of Dr. Arthur M. Baker, formerly a well known physician of Buffalo, who died about 1887.

